# Microbiota in Irritable Bowel Syndrome and Endometriosis: Birds of a Feather Flock Together—A Review

**DOI:** 10.3390/microorganisms11082089

**Published:** 2023-08-15

**Authors:** Noemi Salmeri, Emanuele Sinagra, Carolina Dolci, Giovanni Buzzaccarini, Giulio Sozzi, Miriam Sutera, Massimo Candiani, Federica Ungaro, Luca Massimino, Silvio Danese, Francesco Vito Mandarino

**Affiliations:** 1 Gynecology/Obstetrics Unit, IRCCS San Raffaele Hospital, Vita-Salute San Raffaele University, 20132 Milan, Italy; dolci.carolina@hsr.it (C.D.); buzzaccarini.giovanni@hsr.it (G.B.); candiani.massimo@hsr.it (M.C.); 2 Gastroenterology & Endoscopy Unit, Fondazione Istituto G. Giglio, Contrada Pietra Pollastra Pisciotto, 90015 Cefalù, Italy; emanuelesinagra83@googlemail.com; 3 Gynecology/Obstetrics Unit, Fondazione Istituto G. Giglio, Contrada Pietra Pollastra Pisciotto, 90015 Cefalù, Italy; giulio.sozzi@hsrgiglio.it (G.S.); miriam.sutera@hsrgiglio.it (M.S.); 4 Department of Gastroenterology and Gastrointestinal Endoscopy, IRCCS San Raffaele Hospital, Vita-Salute San Raffaele University, 20132 Milan, Italy; ungaro.federica@hsr.it (F.U.); massimino.luca@hsr.it (L.M.); danese.silvio@hsr.it (S.D.); mandarino.francesco@hsr.it (F.V.M.)

**Keywords:** irritable bowel syndrome, endometriosis, dysbiosis, microbiota, microbiome, gut–brain, pain, female genital tract

## Abstract

Endometriosis and irritable bowel syndrome (IBS) are chronic conditions affecting up to 10% of the global population, imposing significant burdens on healthcare systems and patient quality of life. Interestingly, around 20% of endometriosis patients also present with symptoms indicative of IBS. The pathogenesis of both these multifactorial conditions remains to be fully elucidated, but connections to gut microbiota are becoming more apparent. Emerging research underscores significant differences in the gut microbiota composition between healthy individuals and those suffering from either endometriosis or IBS. Intestinal dysbiosis appears pivotal in both conditions, exerting an influence via similar mechanisms. It impacts intestinal permeability, triggers inflammatory reactions, and initiates immune responses. Furthermore, it is entwined in a bidirectional relationship with the brain, as part of the gut–brain axis, whereby dysbiosis influences and is influenced by mental health and pain perception. Recent years have witnessed the development of microbiota-focused therapies, such as low FODMAP diets, prebiotics, probiotics, antibiotics, and fecal microbiota transplantation, designed to tackle dysbiosis and relieve symptoms. While promising, these treatments present inconsistent data, highlighting the need for further research. This review explores the evidence of gut dysbiosis in IBS and endometriosis, underscoring the similar role of microbiota in both conditions. A deeper understanding of this common mechanism may enable enhanced diagnostics and therapeutic advancements.

## 1. Introduction

The microbiome is a complex ecosystem that harbors trillions of commensals, symbiotic, and even pathogenic microorganisms [1]. It encompasses various regions of the body such as the oral cavity, nares, vagina, outer skin layer, and particularly the gut, serving as the interface between our bodies and the external non-sterile environment [1].

The establishment and modulation of the gut microbiome begin at birth, and its composition becomes highly adaptable and is influenced by various genetic, nutritional, and environmental factors [2,3]. Functioning as a signaling hub, the microbiome integrates environmental inputs with genetic and immune signals, to influence the host’s health status. Indeed, alterations in the composition and function of the gut microbiota can impact intestinal permeability, digestion, metabolism, and immune responses [4,5].

Emerging evidence suggests that the microbiome plays a fundamental role in maintaining host health, influencing various physiological processes, including immune regulation, nutrient metabolism, neuromodulation, and barrier function [6]. A eubiotic gut microbiome contributes to homeostasis by fortifying the epithelial barrier through mucus production, tight junction formation, pathogen exclusion, minimizing inflammation, and regulating the immune system [6,7]. Dysbiosis, which refers to disruption or change in the microbiota qualitative or quantitative composition, leads to deep changes in intestinal barrier function, therefore promoting inflammation and the development of a wide variety of symptoms and diseases [7,8].

As the microbiota and the human body are interconnected components of a single biological system, changes in one element inevitably affect the other components. It is noteworthy that human-origin cells constitute only 10% of the total number of living cells in the human body, while the microbiota represent the remaining 90% of cells [9]. Furthermore, the microbiome encodes over 3 million genes, approximately 150 times more than the total number of genes in the human genome [9]. Therefore, understanding the human microbiome is crucial for comprehending human health and diseases.

Given the mounting evidence regarding the significance of the gut microbiome, it is now well-established that it plays a crucial role in influencing both health and disease [10]. The dynamic nature of the gut microbiome, including its abundance, composition, diversity, and viability, has been found to be responsible for the initiation, development, and treatment of several health disorders [11]. The diversity of microbes within a given body habitat can be defined as the number and abundance distribution of distinct types of organisms, which has been linked to several human diseases; low diversity in the gut to obesity and inflammatory bowel disease, for example, and high diversity in the vagina to bacterial vaginosis [9,10].

The involvement of the human intestinal microbiome has been firmly established in the pathophysiology of various gastrointestinal organic diseases, including colorectal polyps and cancer [12,13], inflammatory bowel diseases [14], and eosinophilic esophagitis [15]. Furthermore, it has been implicated in gastroparesis [16,17] and the occurrence of post-surgical complications [18,19] that necessitate endoscopic treatment [20,21,22,23].

In recent years, there has been a growing focus on elucidating the role of the gut microbiome, not only in gastrointestinal diseases, but also in a broad range of systemic conditions, spanning metabolic disorders such as obesity and type 2 diabetes, to autoimmune diseases, cardiovascular conditions, and even mental health disorders [24,25,26,27].

Dysbiosis in the microbiota has also been suggested to play a role in diseases with partially undisclosed pathogenesis, including two closely related conditions: endometriosis [28,29,30] and irritable bowel syndrome (IBS) [31,32,33]. Several hypotheses have been proposed to link these two diseases, such as hormonal dysregulation, immune system dysfunction, and shared genetic factors. While the precise underlying mechanisms connecting endometriosis and IBS are not yet fully understood, recent studies have implicated an intricate interplay between the microbiota and the host in the pathogenesis of these complex, chronic, and multifactorial disorders. In particular, host–microbiota interactions are known to play a fundamental role in immune system development, thus potentially contributing to the onset, development, and maintenance of immune-mediated diseases and chronic disorders [34], potentially including IBS and endometriosis.

Given the shared characteristics between these conditions, further understanding of the microbiome composition in endometriosis and IBS may offer valuable insights into the unresolved questions regarding their pathogenesis, as well as the common phenotypic and symptomatic traits they present.

Therefore, the objective of this comprehensive review is to provide a detailed scientific overview of the gut microbiome and its potential implications in the development and progression of two complex diseases with currently undisclosed pathogenesis: IBS and endometriosis. By examining recent research findings, this review aims to elucidate the intricate interplay between the gut microbiome and these disorders, offering valuable insights into potential therapeutic approaches for the management of these challenging conditions.

## 2. Microbiota in Irritable Bowel Syndrome

### 2.1. Insights on Irritable Bowel Syndrome

IBS is a chronic disorder characterized by changes in bowel habits, accompanied by abdominal pain or discomfort. It is categorized into four subtypes, based on the primary stool pattern: IBS characterized by diarrhea (IBS-D), IBS with constipation (IBS-C), IBS characterized by a combination of both (IBS-M: mixed), or IBS that cannot be classified into any specific subtype (IBS-U; unclassified) [35].

IBS is classified as a functional gastrointestinal disorder (FGID). However, the concept that symptoms are not linked to structural or metabolic abnormalities [36] has recently been challenged [37].

The diagnosis of IBS is made clinically using the Rome IV criteria, which define IBS as recurrent abdominal pain occurring at least once per week on average over the past three months, along with two or more of the following: symptoms related to defecation, changes in stool frequency, and changes in stool form. These criteria should be met for the past three months, with symptom onset occurring over six months prior to the diagnosis [38].

It is estimated that around 11% of the global population suffers from IBS, with variations in geographical distribution. The lowest prevalence rates have been found in South Asia (7.0%), while the highest rates have been observed in South America (21.0%) [39]. Women are affected more than men, with a ratio ranging from 1.5 to 3 [40,41]. Although IBS can occur in patients of all age groups, symptoms typically begin around the age of 35 in approximately half of individuals [40].

IBS significantly impacts the quality of life and work productivity of affected individuals. It is associated with a noticeable reduction in health-related quality of life [42], an increased likelihood of experiencing psychological comorbidities such as depression and suicidal thoughts [43], high rates of work absenteeism [44], and an increase in medical exams and prescriptions. According to the analysis conducted by Longstreth et al., patients with IBS reported higher rates of cholecystectomy (three-times higher), appendectomy and hysterectomy (twice as high), and back surgery (50% higher) compared to healthy individuals [45]. Consequently, IBS leads to increased costs for both patients and the healthcare system, with estimated direct costs exceeding USD 1 billion in the United States [46].

The pathogenesis of IBS is complex and still being studied for a better understanding. While some studies primary focused on gastrointestinal motor disturbances, such as changes in intestinal transit and abnormal contractions [47], it is now recognized that the pathobiology of IBS is multifactorial. It involves a combination of several factors, including genetic changes [48,49,50], dysregulation of the brain–gut axis, altered central nervous system (CNS) processing, post-infectious changes [51,52], low-grade mucosal inflammation [53,54], immune activation [55], alterations in intestinal permeability [56], and dysbiosis of gut microbiota. In recent years, the relationship between the gut microbiota and IBS has garnered significant interest in both research and clinical settings.

### 2.2. Dysbiosis in IBS

Extensive data have demonstrated significant differences of the gut microbiome composition between individuals with IBS and healthy controls, suggesting that dysbiosis may be involved in the pathogenesis of the disorder. However, the taxonomic characterization of dysbiosis associated with IBS has produced mostly conflicting results, and specific bacterial groups have not been consistently identified. Table 1 shows the main data concerning bacterial dysregulation in dysbiosis related to IBS.

The first study aimed at characterizing the microbiota of IBS patients utilizing a molecular-based approach was conducted in 2007. This study revealed significant differences in the fecal microbiota between IBS patients and healthy controls in several bacterial genera, including *Coprobacillus*, *Collinsella*, and *Coprococcus* [57].

Since then, significant efforts have been dedicated to identifying changes in the gut microbiota associated with IBS [33].

In a study conducted by Jeffery and colleagues, which included 80 IBS patients and 65 healthy controls, an abundance of *Ruminococcus gnavus* and *Lachnospiraceae*, as well as lower levels of *Barnesiella intestinihominis* and *Coprococcus catus* were detected in the fecal microbiota of IBS patients [58]. These findings are consistent with other research that identified a higher abundance of *Ruminococcaceae*, *Bacteroidetes,* and *Lachnospiraceae* in individuals with IBS [59,60,61].

In a meta-analysis of 13 articles, involving 360 IBS patients and 268 healthy subjects, Liu et al. found decreased levels of *Bifidobacterium*, *Faecalibacterium prausnitzii*, and *Lactobacillus* in the fecal microbiota of IBS individuals, particularly those with IBS-D, compared to the control group [62].

**Table 1:** Dysbiosis in the fecal microbiota of patients with irritable bowel syndrome.

Bacteria		Dysregulation	References
***Phyla***	*Firmicutes* to *Bacteroidetes* ratio	↑	[63]
	*Bacteroidetes*	↑↓	[59,60,61,63]
***Species***	*Barnesiella intestinihominis*	↓	[58]
	*Coprococcus*	↓	[57,58]
	*Clostridium*	↑	[63]
	*Ruminococcus*	↑	[58]
	*Lactobacillus*	↓	[64]
	*Bifidobacterium*	↓	[64]
	*Escherichia coli*	↓	[64]
	*Prevotella*	↓	[61]

↑ Increased concentration; ↓ Decreased concentration; ↑↓ Contrasting results.

In a meta-analysis of 16 articles, involving 777 patients, it was found that IBS patients had higher levels of *Firmicutes*, lower levels of *Bacteroidetes*, and an increased *Firmicutes* to *Bacteroidetes* ratio (F/B ratio) in their fecal microbiota compared to controls. Additionally, researchers found higher concentrations of *Clostridia* and *Clostridiales*, as well as lower levels of *Bacteroidia* and *Bacteroidales* at the taxonomic level. However, consistent evidence was not found for the mucosal microbiota [63].

In another meta-analysis of 23 studies involving 1340 subjects, individuals with IBS had lower levels of *Lactobacillus* and *Bifidobacterium*, and higher levels of *Escherichia coli* and *Enterobacter* in their fecal microbiome compared to healthy subjects. No significant differences were found in the levels of fecal *Bacteroides* or *Enterococcus* [64].

In a recent prospective study, Tap and colleagues found that individuals with severe IBS had lower microbial diversity and reduced prevalence of *Prevotella* and *Methanobacteriales.* This microbial signature was able to discriminate between patients with severe symptoms, patients with mild/moderate symptoms, and healthy subjects. Interestingly, the prevalence of *Prevotella* decreased as the severity of symptoms increased [61].

Although several studies identified certain bacteria related to IBS, it is worth noting that significant differences in gut microbiota among different IBS subtypes were not found [60,63].

Alterations in the gut microbiome influence the pathogenesis of IBS through various pathways. First, dysbiosis impacts epithelial permeability, leading to bacterial translocation. This process triggers inflammation and activates both local and systemic immune responses [36]. Locally, cytokines perpetuate microinflammation in the gut, while systemically, pro-inflammatory cytokines such as interleukin-6 (IL-6), tumor necrosis factor-alpha (TNF-alpha), and IL-1β are synthesized [65]. These cytokines have been associated with depression, anxiety, and decreased quality of life [66].

Emerging evidence strongly suggests the existence of a bidirectional communication pathway known as the gut–brain axis, connecting the gut microbiota and central and enteric nervous system [33]. This axis plays a crucial role in regulating various functions, including secretion, immune response, motility, and visceral hypersensitivity [67]. FGIDs, including IBS, are believed to arise from disruptions in the interaction between the gut and brain [68,69,70].

In individuals with IBS, there is a functional neuronal dysregulation, resulting in increased sensitivity within visceral organs, alterations in intestinal motility and changes in immune response. These combined factors contribute to the development of IBS symptoms and the persistence of dysbiosis [33].

Conversely, the gut microbiota can directly modulate brain activity through microbe-generated signals that act on enteroendocrine cells or indirectly through the production of metabolites that interact with afferent vagal and/or spinal nerve endings. It has been found that certain strains of *Clostridia* bacteria modulate intestinal activity by stimulating the biosynthesis and release of serotonin (5-hydroxytryptamine) from enteroendocrine cells [71]. Additionally, the presence of *Clostridia* can disrupt the normal functioning of the gut–brain axis [72]. The altered communication between the gut and the brain can lead to an increased sensitivity to pain signals originating from the intestine. Furthermore, *Clostridia*-induced inflammation and immune activation lead to the generation and maintenance of chronic pain in individuals with IBS.

#### IBS and Small Intestinal Bacterial Overgrowth

Small Intestinal Bacterial Overgrowth (SIBO) is another condition characterized by gut dysbiosis, with proliferation of bacteria in the small intestine, characterized by abdominal pain and alteration of bowel movement. SIBO has been implicated in the pathogenesis of IBS. A meta-analysis of 48 studies found that approximately half of the patients diagnosed with SIBO trough a lactulose breath test and about 1/5 of patients diagnosed through a glucose breath test were also diagnosed with IBS [73]. Recent data suggest that elevated methane gas production can influence intestinal motor activity, leading to slowed intestinal transit and constipation [74,75]. The presence of an excessive number of bacteria in the small intestine can disrupt normal intestinal function and contribute to typical IBS symptoms. However, the relationship between SIBO and IBS is not yet fully understood, and further research is needed to better comprehend this link.

### 2.3. Therapeutical Implications

The role of the microbiota in the pathogenesis of IBS has prompted the exploration of therapeutic measures aimed at improving symptoms by targeting the patients’ microbiota. These interventions include dietary modifications, the use of prebiotics and probiotics, antibiotics (rifaximin), and fecal microbiota transplantation (FMT).

#### 2.3.1. Low FODMAP Diet

More than 50% of patients with IBS report that certain foods worsen their gastrointestinal symptoms [76], and it is widely recognized that diet plays a crucial role in the modulation of the microbiota [77,78].

A low FODMAP (fermentable oligosaccharides, disaccharides, monosaccharides, and polyols) diet is recommended for individuals with IBS [79,80]. FODMAPs are small-chain carbohydrates that are poorly absorbed in the small intestine. Foods containing FODMAPs lead to increased intraluminal water and rapid bacterial fermentation in the colon, resulting in the accumulation of gas and visceral distension. Therefore, eliminating or reducing FODMAPs from the diet could improve the gastrointestinal symptoms of IBS patients.

In a recent meta-analysis of seven randomized controlled trials involving 397 patients, it was shown that low FODMAP diet is associated with a reduction in overall IBS symptoms [81]. The FODMAP diet has been found to be superior to other less restrictive diets, including a low lactose diet [82] and modified NICE diet [83], in terms of alleviating IBS symptoms, particularly abdominal pain and bloating.

The composition of the microbiota could potentially be used to predict the response of IBS patients to the low FODMAP diet. In a trial involving 33 children, it was observed that responders to the low FODMAP diet had higher levels of taxa such as *Bacteroides*, *Ruminococcaceae*, and *Faecalibacterium prausnitzii*, known for their greater saccharolytic metabolic activity [84]. A recent study involving 611 patients with IBS showed that 10 bacterial markers had a positive predictive value of 76% in identifying individuals who would respond positively to the low FODMAP diet [85]. Furthermore, in a randomized controlled trial (RCT) involving 33 IBS patients, it was observed that non-responders to the low FODMAP diet had higher dysbiosis index scores compared to responders at baseline [86].

The low FODMAP diet has been found to have effects on microbiota variation, mostly in the initial “elimination phase”. In the study conducted by Bennet et al., a significant reduction in *Bifidobacterium*, *Actinobacteria*, and *Mycoplasma hominis* was observed after a 4-week trial of low FODMAP diet [86]. Similar findings were reported in the RCT conducted by Staudacher et al., where IBS patients experienced adequate control of symptoms and showed a decreased amount of *Bifidobacteria* in the fecal microbiota after following the low FODMAP for 4 weeks [87].

The reduction in *Bifidobacteria* in fecal microbiota after a short trial of low FODMAP diet is a counterintuitive finding. It is worth noting that these bacteria are already diminished in the microbiota of IBS patients compared to the healthy population. Additionally, it has been found that the increase in *Bifidobacteria* in the gut microbiota following probiotics administration is also associated with an improvement of IBS symptom. Further studies are necessary to comprehend the effects of the low FODMAP diet on the microbiota of patients with IBS [84].

#### 2.3.2. Prebiotics and Probiotics

Prebiotics are non-digestible compounds found in carbohydrates that promote the growth and activity of beneficial gut bacteria.

Prebiotics, such as galactooligosaccharides and fructooligosaccharides, have been shown to increase the levels of *Lactobacilli* and *Bifidobacteria,* which are known to be lower in the gut microbiome of IBS patients [88].

In the RCT conducted by Silk et al., patients with IBS who underwent a 12-week trial of trans-galactooligosaccharide prebiotic exhibited increased levels of *Bifidobacteria* in their fecal microbiota at the end of the treatment compared to the placebo group. The low-dose prebiotic group (3.5 g/dL) reported positive changes in stool consistency, reduced flatulence, and improved overall symptoms and subjective global assessment (SGA) scores. Meanwhile, patients who received prebiotic at high dosage (7 g/dL) showed improvements in SGA and anxiety scores [89].

It has been suggested that prebiotics improve IBS symptoms because they also have anti-inflammatory and antioxidative properties [90]. However, several other studies have reported no significant changes in IBS symptoms following treatment with prebiotics [91,92].

Probiotics are formulations of beneficial bacteria, most commonly *Lactobacillus* and *Bifidobacterium*, that can be delivered and introduced into the gut microbiome. In a meta-analysis of RCTs involving 3452 patients with IBS, it was found that probiotic consumption resulted in a beneficial effect on symptoms of IBS, including abdominal pain, flatulence, and bloating and flatulence [93]. Other meta-analyses reported similar findings [94,95,96,97].

Probiotics could exert their effects on the pathobiology of IBS through several mechanisms. First, they may interact with the regulation of the CNS. In a study conducted by Whang et al., individuals who underwent a 4-week trial of the probiotic strain *Bifidobacterium longum* showed changes in brain activity, with reduced responses in the amygdala and fronto-limbic regions, when exposed to social stressors [98]. It has been hypnotized that these changes may lead to gut–brain axis alterations, involving the processing of serotonin and dopamine [33]. Furthermore, probiotics have been postulated to enhance the functioning of the mucosal barrier and reduce intestinal permeability [96,97]. Additionally, it has also been proposed that probiotics stimulate the synthesis of cytokines, including IL-10, thereby modulating the immune response [99].

While evidence from RCTs indicates benefits in alleviating IBS symptoms, the data are not definitive in identifying the most effective bacterial strains and which IBS variants can benefit from probiotics consumption.

Currently, there are no official recommendations from international societies regarding the use of prebiotics and probiotics in the management of IBS.

#### 2.3.3. Antibiotics

Rifaximin is a commonly used antibiotic in the treatment of IBS. This oral antibiotic has broad-spectrum activity, with minimal absorption into the systemic circulation, making it a favorable choice for gastrointestinal tract infections [100].

In two double-blinded multicenter RCTs, known as TARGET 1 and TARGET 2, IBS patients without constipation were randomly assigned to receive either 550 mg of Rifaximin or a placebo, three times daily for 2 weeks. The results indicated that a higher proportion of patients in the Rifaximin group experienced relief from global IBS symptoms one month after treatment (40.7 vs. 31.7%, *p* = 0.01, when the results of both studies were combined). Moreover, there were no significant differences in terms of adverse events between the Rifaximin and placebo group [101].

A subsequent meta-analysis of five RCTs, which included TARGET 1 and 2 data, confirmed the finding that administering antibiotics is associated with improvements in IBS symptoms [102]. Consequently, major international guidelines recommend the use of Rifaximin in patients with IBS-D [79,80].

The exact mechanism by which Rifaximin acts on IBS symptoms is not fully understood. It has been postulated that Rifaximin may modulate the gut microbiota, reducing the burden of bacteria responsible for micro-inflammation and immune responses [101]. However, in the TARGET 3 study, the fecal microbiota of IBS patients treated with Rifaximin showed only modest and temporary changes. Furthermore, these effects were mostly reversed at the end of the study, 46 weeks after treatment [103]. Similar findings were observed in the study conducted by Zeber Luckeka et al., where limited differences in the fecal microbiota were found before and after Rifaximin treatment through metagenomic and metabolomic analysis [104].

Further data will be needed to better understand the effects of Rifaximin on the composition of the gut microbiota.

#### 2.3.4. Fecal Microbiota Transplantation

FMT involves transferring fecal material from a healthy donor into the gastrointestinal tract of other subjects, to modify their gut microbiota. While FMT has been extensively researched as a safe and effective treatment for *Clostridium difficile* infection [105], its potential as a treatment for IBS has only recently been explored.

In a double-blind placebo-controlled study, 165 patients with IBS were randomized to receive placebo (autologous feces), 30 g FMT or 60 g FMT administered via gastroscope. Patients who received 30 g and 60 g FMT experienced a significant improvement of IBS symptoms compared to placebo (76.9%, 89.1%, 23.6%, respectively) [106]. Other robust data from RCTs have also shown that FMT via colonoscope or gastroscope is associated with a significant improvement in IBS symptoms [107,108]. However, in the double-blinded RCT conducted by Madsen et al., FMT administered via capsules for 12 days did not significantly improve abdominal pain, stool frequency, or stool form in patients with moderate-to-severe IBS up to six-month follow up [109]. Another meta-analysis of 5 RCTs involving 267 patients found that IBS symptoms did not significantly improve after FMT [110].

Several studies have also examined changes to the gut microbiome after FMT.

El-Salhy and colleagues found an increased concentration of *Lactobacilli*, *Eubacterium biforme*, and *Alistipes* and a reduced concentration for *Bacteroides* in microbiota in the fecal microbiota of IBS patients one month after FMT. Notably, the concentration of *Alistipes* and *Lactobacillus* correlated with the IBS-SSS score, suggesting that the modulation in gut composition may lead to clinical changes [106]. In the study of Halkajer et al., the microbiome of patients with IBS who received FMT capsules did not differ from the donors’ microbiota at the end of treatment [111]. In the study conducted by Mazzawi et al., significant changes in *Ruminococcus gnavus*, *Actinobacteria,* and *Bifidobacteria* were observed 3 weeks after FMT. However, significant levels of *Bifidobacteria* and *Actinobacteria* decreased by the 20th week [112].

Further data are needed to clinically correlate changes in gut microbiome composition with symptomatic relief in IBS patients.

## 3. Microbiota in Endometriosis

### 3.1. Insights on Endometriosis

Endometriosis is a chronic, estrogen-dependent inflammatory condition characterized by the presence of endometrium-like tissue outside the uterus [113]. It affects approximately 10% of women during their reproductive years, corresponding to around 190 million women worldwide [114]. The disease imposes a considerable health burden, resulting in a lifetime cost of USD 27,855 per year per patient [115]. This substantial financial burden is attributed to the expenses associated with treatment, work loss, and overall healthcare costs. Diagnosis of endometriosis typically involves laparoscopy, contributing to an average diagnostic delay of seven years after the onset of symptoms [116].

Histopathologically, endometriotic lesions within the abdominal cavity are classified as ovarian endometrioma (OMA), deep endometriosis (DE), and superficial peritoneal endometriosis (SPE) [117]. Macroscopically, the disease can be found in various pelvic locations, including but not limited to the pelvic peritoneum, ovaries, bladder, rectovaginal septum, and gastrointestinal tract. Extra-abdominal endometriosis has also been reported [118]. The multifocality of the lesions contributes significantly to the complex clinical presentation of endometriosis, posing a significant challenge in its management [119].

Key clinical features of endometriosis include debilitating pelvic and abdominal pain, accompanied by dysmenorrhea (painful menstruation), dyspareunia (painful sexual intercourse), dyschezia (pain during defecation), and dysuria (pain during urination) [113,114]. Infertility is more prevalent in patients with endometriosis, doubling the risk compared to women without the condition [120].

Notably, conventional treatments for endometriosis-related symptoms, such as surgery or hormonal therapies, have demonstrated efficacy in symptom management [121,122,123,124] and for improving fertility outcomes [125,126]; however, treatment response varies, and long-lasting symptom relief is not consistently achieved.

This observation aligns with the growing understanding of endometriosis as a multisystem condition characterized by diverse genetic and somatic traits [127,128], along with a significant association with various comorbidities, primarily autoimmune [129] and immune-related diseases [130], but also fibromyalgia [131], migraine [132], and multiple pregnancy-related disorders [133,134,135]. The etiology of these comorbidities, whether resulting from a shared pathogenesis or the chronic inflammatory response to the endometriotic lesions, remains unknown [136], although the severity of endometriosis is known to increase with the co-occurrence of comorbidities [137,138,139].

In particular, women diagnosed with endometriosis have a two to threefold increased risk of fulfilling the complete criteria for IBS, and more than 20% of women with endometriosis experience symptoms resembling IBS [140]. Notably, gastrointestinal symptoms, including abdominal bloating, diarrhea, or constipation, significantly impact the quality of life of women with endometriosis, even in the absence of macroscopic intestinal endometriosis lesions [141].

Despite notable advancements in medical research, the pathogenesis of endometriosis remains largely elusive, with a large variety of postulated genetic, metabolomic, immunology, endocrinology, and environmental factors [142,143,144]. Therefore, investigating and evaluating associated symptoms can offer valuable insights, and leveraging existing knowledge from other diseases and comorbidities can enhance our understanding of the underlying etiopathogenetic mechanisms involved in endometriosis.

The shared symptomatology and frequent co-occurrence of endometriosis and IBS have prompted investigations into their biological relationship, exploring aspects such as immunological factors, hormonal imbalances, and visceral hypersensitivity [145,146]. Moreover, mounting evidence strongly suggests the crucial involvement of the gut–brain axis in the etiopathogenesis of endometriosis, underscoring the potential of microbiomes and dysbiosis as novel therapeutic targets, not only for alleviating gastrointestinal symptoms, but also for addressing the diverse range of pain and symptoms associated with endometriosis [147].

### 3.2. Dysbiosis in Endometriosis

The cutting-edge “genetic-epigenetic theory” proposed by Koninkx and colleagues [148,149] has led to the extensive investigation of factors related to the genetic-epigenetic cellular events underlying endometriosis onset and development, including immunologic, endocrine, paracrine, and microbiotal factors. In particular, dysbiosis has been extensively demonstrated in patients with endometriosis and animal models of the disease [28,30,147,150,151,152]. Eubiosis is characterized by high levels of *Firmicutes* and *Bacteroidetes* (>90%) and a low percentage of *Proteobacteria*, while dysbiosis is linked to an altered F/B ratio [153]. Animal studies have consistently shown an increased F/B ratio associated with endometriosis, ranging up to two-fold in most cases [154,155], although some studies have reported conflicting results [156]. In humans, F/B ratio and microbial diversities have been less explored, with only one study on human fecal samples showing a lower α diversity of gut microbiota and a higher F/B ratio in severe endometriosis cases compared to controls [157]. Nevertheless, a common observation is a reduced overall microbial diversity within the gut of women with endometriosis [158]. Table 2 displays a characterization of dysbiosis in endometriosis.

A systematic review published in 2019 found that endometriosis is associated with an increased presence of *Proteobacteria, Enterobacteriaceae, Streptococcus* spp., and *Escherichia coli* across various microbiome sites [28]. The phylum *Firmicutes* and the genus *Gardnerella* also showed some associations, albeit with conflicting results [28]. Among other microbiota alterations suggested to be linked with endometriosis, independent studies found significant increases in *Actinobacteria, Cyanobacteria, Saccharibacteria, Fusobacteria, Acidobacteria*, and *Patescibacteria* [154,155,157]. A consistent finding in both animal and human studies is the higher concentration of Gram-negative bacteria in endometriosis [157,158,159,160]. Notably, species belonging to the phyla *Proteobacteria, Bacteroidetes*, and *Negativicutes,* characterized by Gram-negative staining, particularly the genera *Shigella* and *Escherichia,* as well as the *Prevotella* species, were significantly increased in endometriosis cohorts in both intestinal and cervicovaginal or intrauterine sampling [160,161]. These alterations were especially pronounced in patients with gastrointestinal symptoms such as constipation, bloating, flatulence, vomiting, and nausea [158]. Protective microbes in the gut of women with endometriosis have also been found to be diminished. Huang et al. reported reduced abundances of *Clostridia, Ruminococcus*, and *Lachnospiraceae* at the genus level, which are commensals known to produce short-chain fatty acids (SCFAs) that regulate intestinal integrity and are implicated in various diseases associated with gut–microbiome dysbiosis [162]. Interestingly, hormonal treatment for endometriosis has been shown to increase *Ruminococcus* and other SCFA producers in patients [158]. Additionally, the genera *Sneathia, Barnesella,* and *Gardnerella* were significantly reduced, particularly in advanced stage 3/4 endometriosis cases [160].

**Table 2:** Dysbiosis in fecal microbiota of patients with Endometriosis.

Bacteria		Dysregulation	References
***Phyla***	*Firmicutes* to *Bacteroidetes* ratio	↑	[154,155]
	*Bacteroidetes*	↑	[160,161]
	*Proteobacteria*	↑	[160,161]
***Species***	*Escherichia coli*	↑	[160,161]
	*Streptococcus*	↑	[28]
	*Gardnerella*	↑↓	[28,160]
	*Clostridium*	↓	[162]
	*Ruminococcus*	↓	[162]
	*Prevotella*	↑	[160,161]

↑ Increased concentration; ↓ Decreased concentration; ↑↓ Contrasting results.

The precise alterations in the microbiome related to endometriosis are still under investigation. However, the significance of these changes is supported by the existence of several proposed mechanisms through which the gut microbiota influences endometriosis. To comprehend the intricate bidirectional relationship between the microbiome and endometriosis, various mechanisms come into play, including microbial-induced inflammation and immune dysregulation, alterations in estrogen metabolism, and the modulation of pain pathways through the gut–brain axis.

First, it has been suggested that the influence of the microbiome on immunomodulation and the development of chronic inflammation in endometriosis could be crucial for the maintenance and progression of the disease [163]. Indeed, microbiome-dependent immune homeostasis guarantees that the immune system remains tolerant to its self-components, commensals, and foods, and reactive to pathogens, to prevent external insults and bacterial translocation [164]. Dysbiosis increases inflammation in the intestinal epithelium, increases permeability, and ultimately disrupts barrier function, causing immune imbalance and low-grade systemic inflammation [164,165]. According to the bacterial contamination hypothesis, microbial pathogens can activate the host immune response by binding with toll-like receptors (TLRs) [165]. In particular, Gram-negative bacteria such as *Proteobacteria*, which were found to be higher in endometriosis, contain in their cell wall an enterotoxin called lipopolysaccharide (LPS) that promotes inflammation through binding to toll-like receptor-4 (TLR-4), contributing to the onset and progression of endometriosis lesions [164,166,167]. The binding between lipopolysaccharide and TLR-4 significantly increases the concentration of peritoneal cavity immune cells, especially macrophages [168], which produce TNF-alpha, IL-1 receptor, vascular endothelial growth factor (VEGF), IL-6, IL-8, and IL-17, and which can promote the formation, infiltration, and neoangiogenesis of endometriotic peritoneal nodules [161,164,165,166,167,168,169]. Therefore, in endometriosis, dysbiosis-mediated intestinal inflammation contributes to enhancing the dysregulated immune response observed in the disease, ultimately creating an immunosuppressive environment that enables the spread and growth of escaped ectopic endometrial cells outside the uterus [163].

Another theory that elucidates the microbiome’s role in contributing to the development of endometriosis is its impact on estrogen metabolism [170]. The gut microbiota functions as a comprehensive endocrine organ, exerting diverse effects on the intestinal environment, which in turn influences distant organs and pathways. Throughout a woman’s lifetime, the gut microbiota plays a significant role in the reproductive endocrine system by interacting with hormones such as estrogen, androgens, insulin, and others. The gut microbiota houses the “estrobolome”, which encompasses the gene inventory responsible for encoding estrogen-metabolizing enzymes [171]. Notably, an analysis of microbial genomes revealed that various genera within the gut microbiome encode for the production of β-glucuronidase, including *Bacteroides, Bifidobacterium, Streptococcus, Escherichia,* and *Lactobacillus* [172]. Estrogens undergo metabolism from their conjugate forms to their deconjugated forms through microbial-secreted β-glucuronidase, glucosidases, and hydroxysteroid dehydrogenases [173]. The resulting free estrogens, deconjugated from glucuronic acid by bacterial enzymatic action, are absorbed into the circulatory system as active estrogen and act on estrogen receptors in the body [141,154]. Therefore, alterations in the gut microbial composition and β-glucuronidase activity could potentially perturb or dysregulate circulating estrogen levels, leading to hyper-estrogenic states and contributing to estrogen-mediated conditions, such as endometriosis [174]. Additionally, the link between microbiota and estrogen metabolism is closely intertwined with inflammation. The enhanced levels of β-glucuronidase expression observed in endometriosis lesions compared to the normal endometrium promote endometriosis progression by stimulating the proliferation and migration of endometrial stromal cells. This effect is mediated both directly and indirectly through macrophage M0 to M2 polarization, leading to an immune imbalance in vitro model from human samples and in mice models [175].

The microbiome has been suggested to play a critical role in modulating pain pathways through the gut–brain axis [176]. This bidirectional communication is thought to contribute to the central sensitization of chronic pain by regulating neuroinflammatory responses [176]. Specifically, the microbiome influences the activity of microglia and astrocytes, leading to increased glutamate levels and decreased gamma-amino-butyric acid (GABA) levels in central synaptic neurotransmission, ultimately resulting in pain hypersensitivity [177]. Interestingly, central sensitization is known to be significantly involved in endometriosis-associated chronic pelvic pain [178]. Thus, the role of the gut microbiota in neuroinflammation, which contributes to central sensitization, may also underlie the chronic pain experienced in endometriosis. Dysbiosis, or an imbalance in the gut microbiome, could potentially lead to incorrect immune responses, triggering the development of inflammatory pain, such as that seen in endometriosis [179]. Similarly, the chronic visceral pain associated with functional gastrointestinal disorders such as IBS may also result from disruptions in the gut microenvironment.

**Figure 1:**
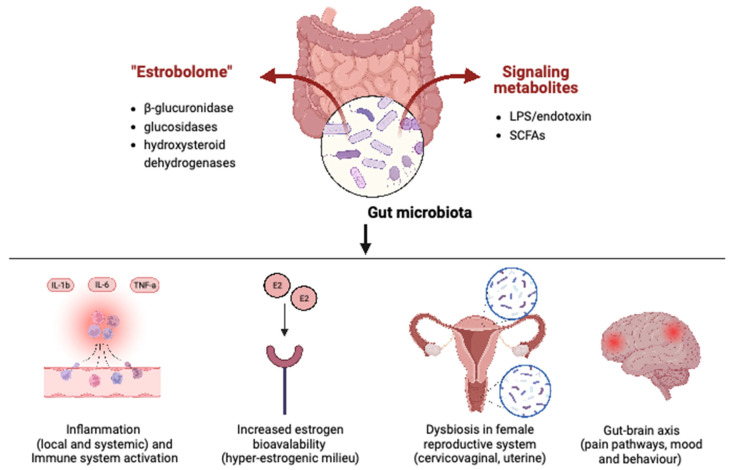
Mechanisms of gut microbiota involvement in endometriosis and IBS. Abbreviations: LPS, lipopolysaccharide; SCFAs, short-chain fatty acids; IL-1b, interleukin-1b; IL-6, interleukin-6; TNF-a, tumor necrosis factor alpha; E2, estrogens.

Figure 1 provides an overview of the mechanisms by which the gut microbiome is involved in both endometriosis and IBS.

#### Female Genital Tract Microbiome

The urogenital microbiota, also known as the female genital tract (FGT) microbiome, constitutes approximately 9% of the total microbiota and contains around 108 UFC per gram of vaginal mucus [180]. While the gut microbiota is highly diverse, the vaginal microbiome typically exhibits low diversity within each individual, with a predominance of Lactobacillus species in most healthy white premenopausal women [181]. *Lactobacilli* play a protective role in the cervicovaginal environment by producing lactic acid, hydrogen peroxide, and bacteriocins, which maintain an acidic ecosystem with a pH between 3.5 and 4.5, unfavorable for the growth of other bacteria [182]. Additionally, *Lactobacillus* spp. contribute to homeostasis by occupying this niche (pathogen exclusion) and producing anti-inflammatory cytokines and antimicrobial peptides from epithelial cells, fortifying the epithelial cell barrier [183]. A *Lactobacillus*-deficient cervicovaginal system is correlated with higher concentrations of genital pro-inflammatory cytokines and increased activation of antigen-presenting cells (APCs) through LPS pathways [184].

In women with endometriosis, the vaginal microbiota composition was found to show a decrease in the abundance of *Lactobacillus* species and higher abundance of *Anaerococcus* compared to controls [185]. However, the results were contradictory, as other studies found no differences in the vaginal microbiome compared to healthy controls [186,187]. The uterine microbiota may also be related to endometriosis and infertility. Although the debate is ongoing, advancements in 16S RNA detection methods have given rise to the in-utero colonization hypothesis, challenging the sterile womb hypothesis [188]. The colonization of the upper FGT microbiome is suggested to have various origins through different routes, including ascendant pathways from the vagina or vulva, bloodstream or lymphatic transport from intestinal microbiota after crossing the bowel wall, or even from the oral cavity [189]. Despite recent discoveries providing evidence for a nonsterile endometrium, the presence and characterization of a resident endometrial microbiome remain elusive [190,191]. Nonetheless, evidence suggests that colonization of the endometrium by dysbiotic bacteria or the lack of *Lactobacillus* dominance in the lower genital tract could negatively impact fertility, a key characteristic of endometriosis [192]. Furthermore, studies assessing the microbial composition of the FGT in patients with endometriosis found that cervicovaginal and sometimes uterine microbiota in endometriosis was characterized by non-*Lactobacillus* dominance, with enrichment of a variety of opportunistic pathogens and bacterial species commonly observed in bacterial vaginosis (BV), including *Enterobacteriaceae*, *Streptococcus*, *Pseudomonas*, *Corynebacterium*, *Streptococcus*, *Gardnerella*, *Escherichia*, *Shigella*, and *Ureaplasma* [187,193,194]. This suggests that BV-associated organisms may also be consistently associated with endometriosis.

Importantly, whether changes in the microbiome of the FGT and/or gastrointestinal tract occur simultaneously and the exact contribution of dysbiosis in different locations to endometriosis pathogenesis need to be clarified. Nonetheless, there is a correlation in the microbial composition of both intestinal and cervicovaginal microbial niches, with an over 50% overlap in species presence and cell density per bacterial species [195]. Incorporating this idea of a direct crosstalk between the gut and the FGT may help clarify the role of alterations in the microbiome composition of the gut and FGT in women with endometriosis.

### 3.3. Therapeutical Implications

A mounting body of evidence points to microbiota imbalances in various diseases, suggesting that restoring eubiosis could be a viable treatment option for several non-FGID conditions [196]. Exploring the role of the intestinal and cervicovaginal microbiome in endometriosis opens significant opportunities for developing innovative diagnostic and therapeutic strategies for the disease.

Recent research has proposed that small molecular metabolites derived from gut microbiota could serve as potential diagnostic markers for endometriosis [197]. This advancement may lead to non-invasive diagnostic methods using stool metabolites to detect endometriosis. However, despite the potential for groundbreaking developments in microbiome-related biomarkers to diagnose endometriosis earlier in its progression and predict treatment response, this area remains a subject of hopeful anticipation.

**Figure 2:**
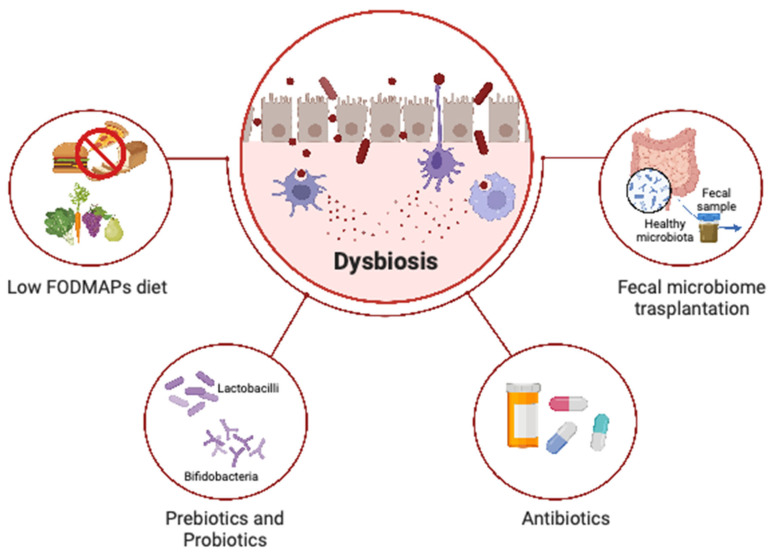
Overview of treatments targeting dysbiosis in endometriosis and IBS.

Furthermore, targeting the gut microbiota in endometriosis holds great promise as a more active and promising field of research. Approaches focused on modulating the gut microbiota, such as dietary interventions, probiotics, prebiotics, or fecal microbiota transplantation, show potential as adjunct therapies to alleviate symptoms and improve outcomes in women with endometriosis [29]. Figure 2 provides an overview of treatments targeting dysbiosis in endometriosis and IBS.

#### 3.3.1. Low FODMAP Diet

A variety of alternative diets, including the low FODMAP diet [198], have been proposed for endometriosis, with particular interest sparked by their success in managing symptoms of IBS and their potential application in conditions associated with gut dysbiosis, such as endometriosis.

One study has demonstrated the effectiveness of the low FODMAP diet in women with gut symptoms and endometriosis [199], showing over 50% improvement in bowel symptoms after four weeks on the diet. It is possible that the reduction in FODMAP intake not only decreases visceral hypersensitivity but also directly affects the composition of the gut microbiome and related inflammatory pathways, potentially leading to improved gastrointestinal symptoms and alleviated abdominal pain associated with endometriosis.

Additionally, evidence suggests that certain diet components, particularly those deficient in vitamin A, C, D, and E, calcium, folate, and beta-carotene but rich in fats and sugar, may cause vaginal dysbiosis [200]. Therefore, implementing these diet components in endometriosis management may also contribute to maintaining a healthy cervicovaginal microbiome, which could potentially benefit disease progression or at least symptom management.

#### 3.3.2. Prebiotics and Probiotics

Numerous animal studies have demonstrated the potential beneficial effects of probiotics, especially *Lactobacillus* spp., in reducing endometriotic lesions by increasing IL-12 concentration and natural killer (NK) cell activity in mice [201]. Furthermore, in rats, probiotics have shown promise in preventing the growth of new endometriosis lesions [202]. In humans, two recent randomized, placebo-controlled trials have provided encouraging evidence that oral administration of *Lactobacillus gasseri* can improve endometriosis-associated pain. The treatment led to a significant reduction in pain intensity measured on the visual analog scale (VAS) and dysmenorrhea on the verbal rating scales (VRS) after an 8-week treatment period [203,204].

Despite these promising results, there is currently a lack of guidelines outlining or supporting the standard use of probiotics in the management of endometriosis. Further research and larger clinical trials are needed to establish their actual efficacy, before probiotics can be widely recommended in the management of endometriosis.

#### 3.3.3. Antibiotics

Antibiotics have emerged as a promising approach for treating endometriosis. Studies in animal models have demonstrated the efficacy of broad-spectrum antibiotic treatments in inhibiting ectopic lesions and reducing the size and weight of endometriotic lesions, accompanied by a significant reduction in inflammatory markers in the peritoneal fluid [156]. Similarly, human studies using specific antibiotics, such as levofloxacin, have shown their ability to reduce tissue inflammation, cell proliferation, and angiogenesis in both eutopic and ectopic endometrium [205]. These findings are of particular significance as they establish a connection between endometriosis and chronic endometritis, thereby presenting new possibilities for novel antibiotic treatment strategies against endometriosis.

Chronic endometritis has been reported to be identified in at least 3–5% of patients with endometriosis, and its prevalence may be underestimated [206]. This indicates that the variety of antibiotics currently employed as first and second-line treatments for endometritis could potentially be suitable for treating endometriosis as well [207].

In a recent study involving 155 women in Japan, members of the bacterial genus *Fusobacterium* were detected in the uteruses of approximately 64% of those with endometriosis. Molecular findings from this study suggested that *Fusobacterium* infection of endometrial cells triggered the activation of transforming growth factor-β (TGF-β) signaling pathways and led to a phenotypic transition of endometrial fibroblasts. This interesting discovery was followed by experiments on mice infected with *Fusobacterium*, where antibiotic treatment was shown to reduce the number and weight of established endometriotic lesions. This finding may offer a potential strategy for managing the fibrotic remodeling caused by chronic inflammation in endometriosis [208].

However, the applicability of antibiotic treatments in endometriosis remains a subject of debate, due to the potential side effects of prolonged use, including alterations in microbial community profiles and lasting disruptions to healthy microbiotas [209]. Further research and clinical studies are needed to fully understand the benefits and risks associated with antibiotic therapy for endometriosis.

#### 3.3.4. Fecal Microbiota Transplantation

An innovative study in mice revealed fascinating findings, demonstrating that either a 21-day regimen (once every 3 days) of vaginal administration of antibiotics or a vaginal microbiota transplantation (VMT) effectively reduced the volume of endometriotic lesions through the regulation of the nuclear factor-kappa B signaling pathway [210].

Additionally, FMT has been proposed as an effective strategy for restoring the gut microbiota and treating various diseases, including female reproductive tract conditions [211]. These promising results suggest that FMT could serve as an innovative and effective treatment option for endometriosis or, at the very least, for alleviating endometriosis-related symptoms, by targeting microbiome restoration and avoiding the potential adverse effects of antibiotics [212].

However, despite the encouraging outcomes, further extensive research is essential to determine the efficacy, safety, and long-term effects of these microbiome-targeted interventions in endometriosis, before translating them into clinical practice.

## 4. Current Limitations and Future Directions

Recent advances in multiomic technology have revolutionized our understanding of microbial communities through various approaches, such as amplicon sequencing, shotgun metagenomic sequencing, and next-generation RNA sequencing. These powerful tools have allowed us to detect alterations in microbial gene expression and uncover functional disease-related profiles in the human microbiome [213]. Despite the challenges in causal discovery within observational microbiome data, methodological improvements in causal structure learning, along with the integration of multiple omics data (such as metatranscriptomics and metabolomics) with metagenomics, have enhanced our ability to predict causal effects in large-scale biological systems [214]. Hierarchical models that incorporate existing biological knowledge about potential system variable relationships have also been suggested to improve the precision of causal discovery. Confirming causative relationships between microbes and disease onset or progression holds significant clinical implications [215]. Longitudinal assessments of the microbiome in the gut and female reproductive tract, with and without concurrent interventions, offer great potential for understanding the underlying causes of these complex diseases, developing new therapies, and finding preventive measures [216].

In the context of IBS and endometriosis, a deeper understanding of functional disease-related microbiome profiles could provide insights into disease-specific phenotypes and the overlapping traits observed in both conditions. Patients affected by these diseases may exhibit distinct or even contrasting clinical manifestations, such as pain-related symptoms versus infertility in endometriosis, and diarrhea versus constipation in IBS.

Deciphering the microbiome’s findings will not only enhance our current understanding of the pathogenesis of these diseases but also shed light on the presence or absence of specific comorbidities, allowing for further stratification of disease-specific subgroups. This knowledge will also aid in identifying the patient population most likely to benefit from microbiome-targeting treatments, including probiotics, prebiotics, and the emerging field of fecal transplantation.

## 5. Conclusions

As the prevalence of IBS and endometriosis continues to increase, there is a growing concern about the escalating economic and social costs associated with these diseases. In response, there is an urgent need for innovative management strategies that go beyond standard care.

One promising avenue for further exploration is the biological basis of these diseases. Advances in high-throughput microbial genomic sequencing and other systems biology techniques have provided valuable insights into the potential role of the gut microbiota in both health and disease. Consequently, a growing number of diseases, including IBS and more recently endometriosis, have been characterized by distinctive changes in the composition and functionality of the gut microbiota. This area of research is currently one of the most exciting fields, as it holds the potential to unravel the pathogenesis of these complex diseases and improve preventive strategies, early diagnosis, effective management, and progression prevention.

However, the question of whether microbiota changes are a cause, consequence, or incidental factor in these diseases remains largely uncertain. Understanding the relationship between microbiome imbalance and disease development is crucial. Does an imbalance in the microbiome precipitate complex diseases such as endometriosis and IBS, or is it a byproduct of the disease state or merely an incidental factor? Only by addressing this critical question can we truly unlock the potential of microbiota-based interventions for managing, treating, and perhaps even preventing these debilitating conditions.

## Data Availability

Not applicable.
